# Supplementary figures and images for: Impact of influenza vaccination on amoxicillin prescriptions in older adults: A retrospective cohort study using primary care data
Source: PLoS One. 2021 Jan 29;16(1):e0246156. doi: 10.1371/journal.pone.0246156 (PMC7846013; doi:10.1371/journal.pone.0246156)

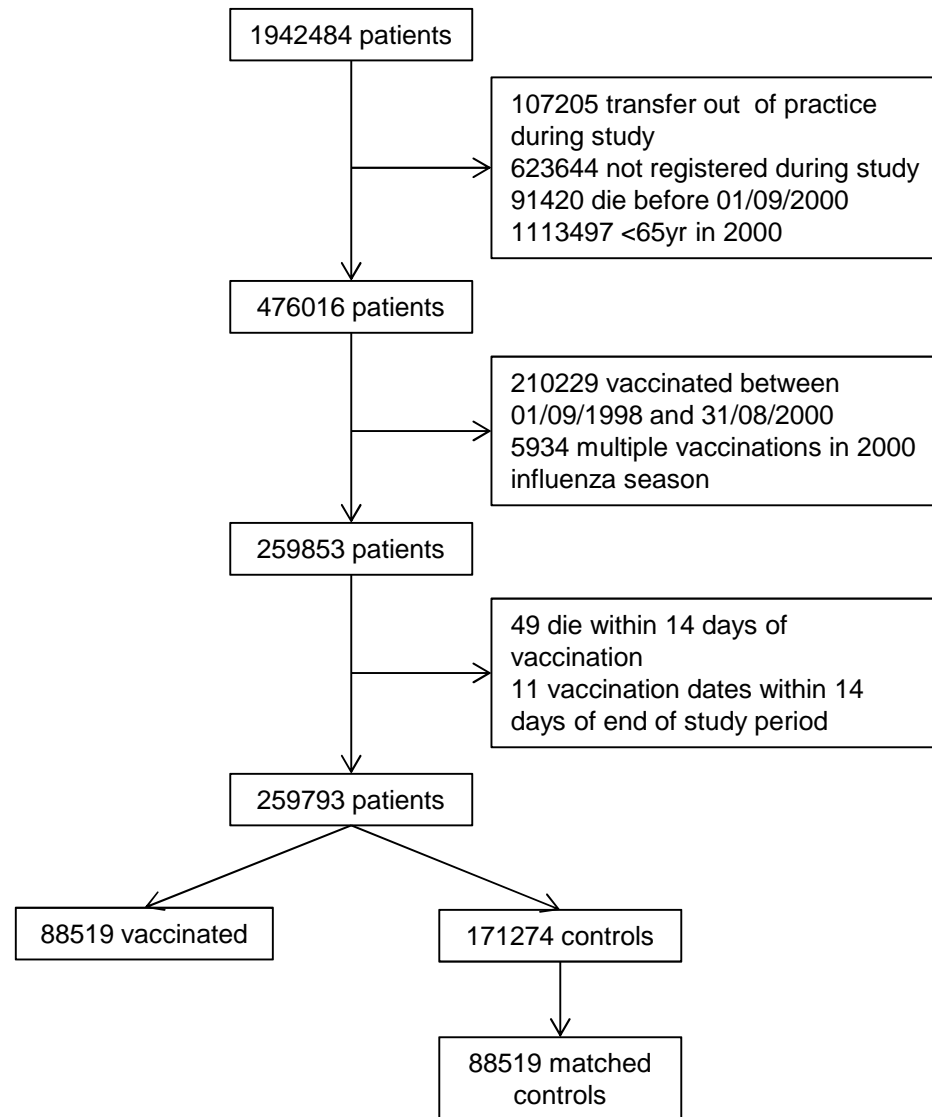

Supplement: S1 Fig — (PDF) [file pone.0246156.s002.pdf]

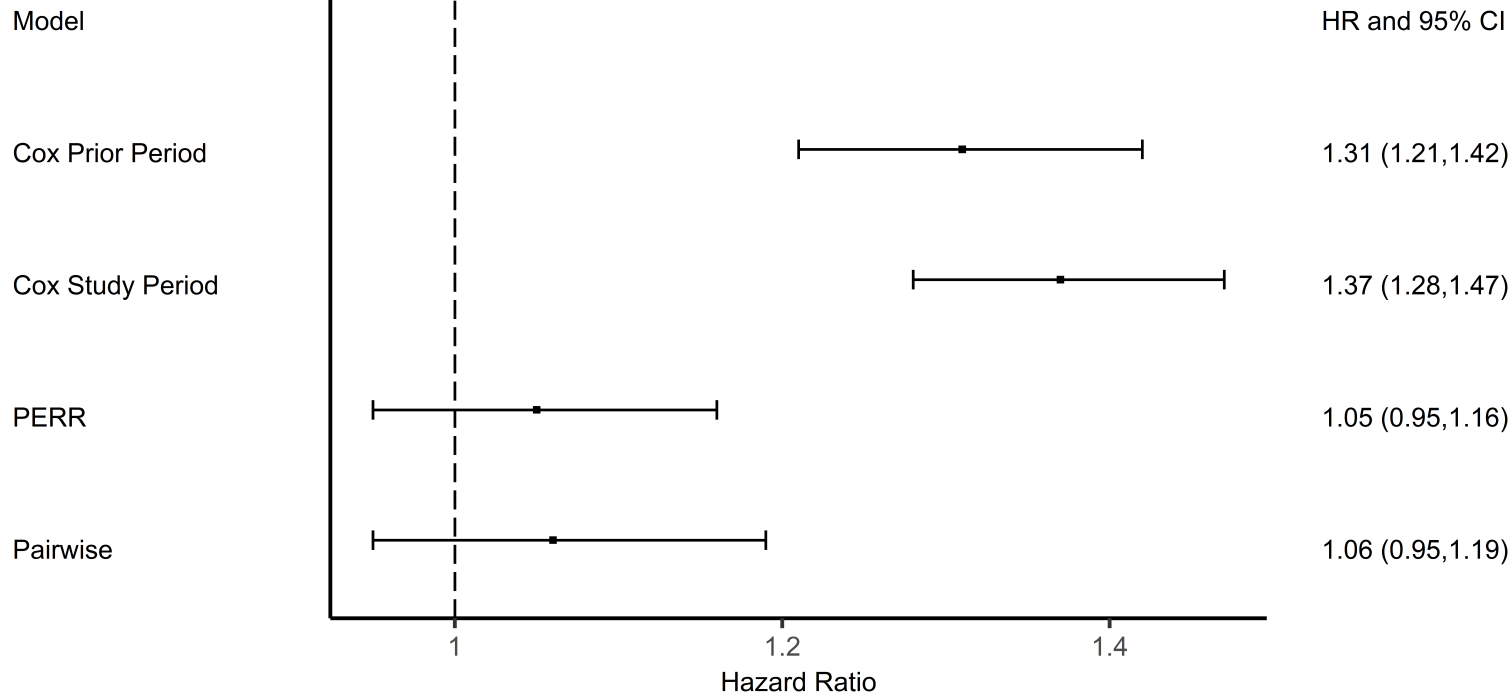

Supplement: S2 Fig — (PDF) [file pone.0246156.s003.pdf]

(1) 0%

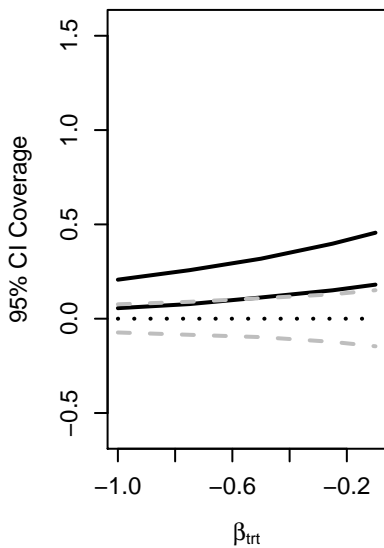

(2) 5%

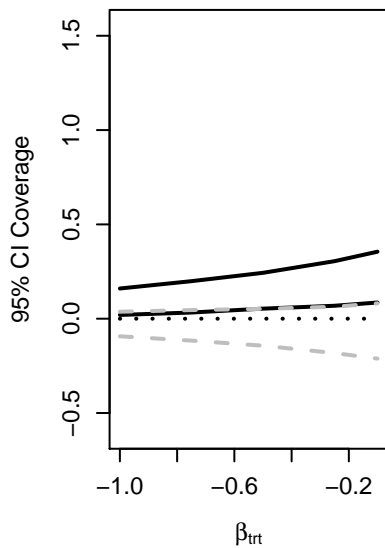

(3) 25%

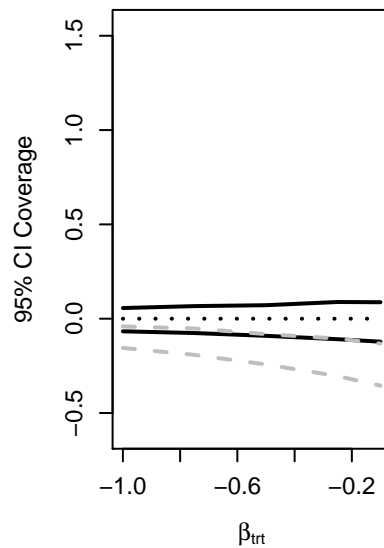

(4) 35%

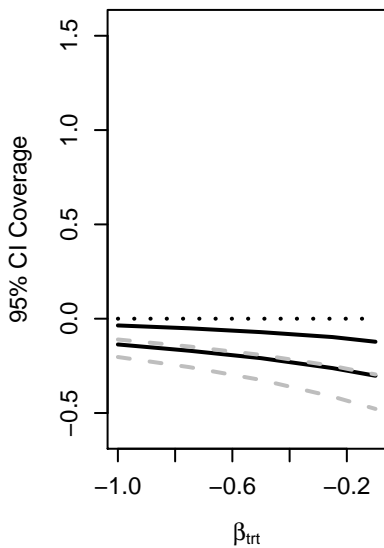

(5) 50%

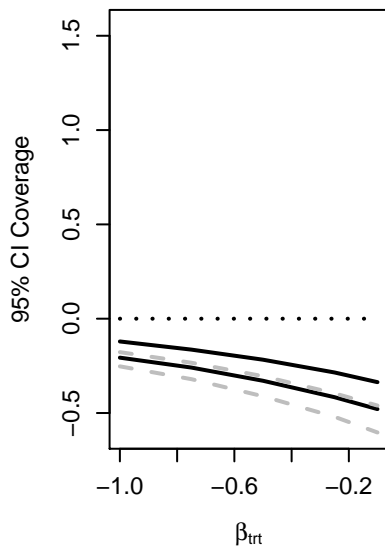

— Cox model  
- - Pairwise  
... True value

Supplement: S5 Fig — Percentage labels indicate the percentage of patient who do not respond to the vaccination e.g. 50% half of the treated patients do not respond to the vaccine. 95% Confidence interval coverage of assessing change in continuous confounder distribution (an interval containing zero covers the true treatment effect). (PDF) [file pone.0246156.s006.pdf]
