# Supplementary material for: Impact of influenza vaccination on amoxicillin prescriptions in older adults: A retrospective cohort study using primary care data
Source: PLoS One. 2021 Jan 29;16(1):e0246156. doi: 10.1371/journal.pone.0246156 (PMC7846013; doi:10.1371/journal.pone.0246156)
Supplement: S3 Fig — 95% Confidence interval coverage of assessing change in continuous confounder distribution (an interval containing zero covers the true treatment effect). (PDF) [file pone.0246156.s004.pdf]

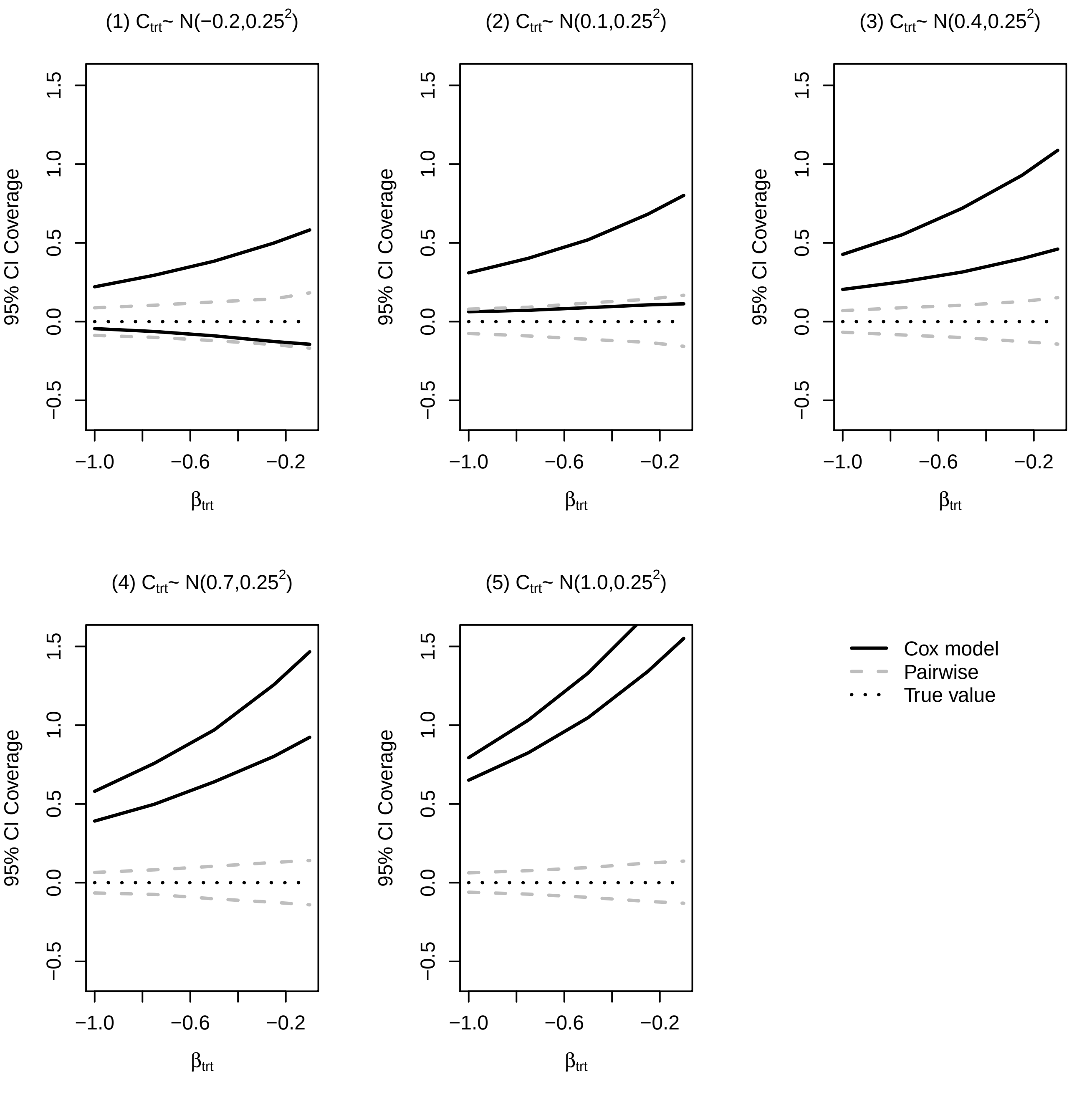
(1) C_{trt}~ N(−0.2,0.25^2)
(2) C_{trt}~ N(0.1,0.25^2)
(3) C_{trt}~ N(0.4,0.25^2)
(4) C_{trt}~ N(0.7,0.25^2)
(5) C_{trt}~ N(1.0,0.25^2)
95% CI Coverage
β_{trt}
−0.5
0.0
0.5
1.0
1.5
−1.0
−0.6
−0.2
Cox model
Pairwise
True value
