# Supplementary material for: Impact of influenza vaccination on amoxicillin prescriptions in older adults: A retrospective cohort study using primary care data
Source: PLoS One. 2021 Jan 29;16(1):e0246156. doi: 10.1371/journal.pone.0246156 (PMC7846013; doi:10.1371/journal.pone.0246156)
Supplement: S4 Fig — 95% Confidence interval coverage of assessing change in continuous confounder distribution (an interval containing zero covers the true treatment effect). (PDF) [file pone.0246156.s005.pdf]

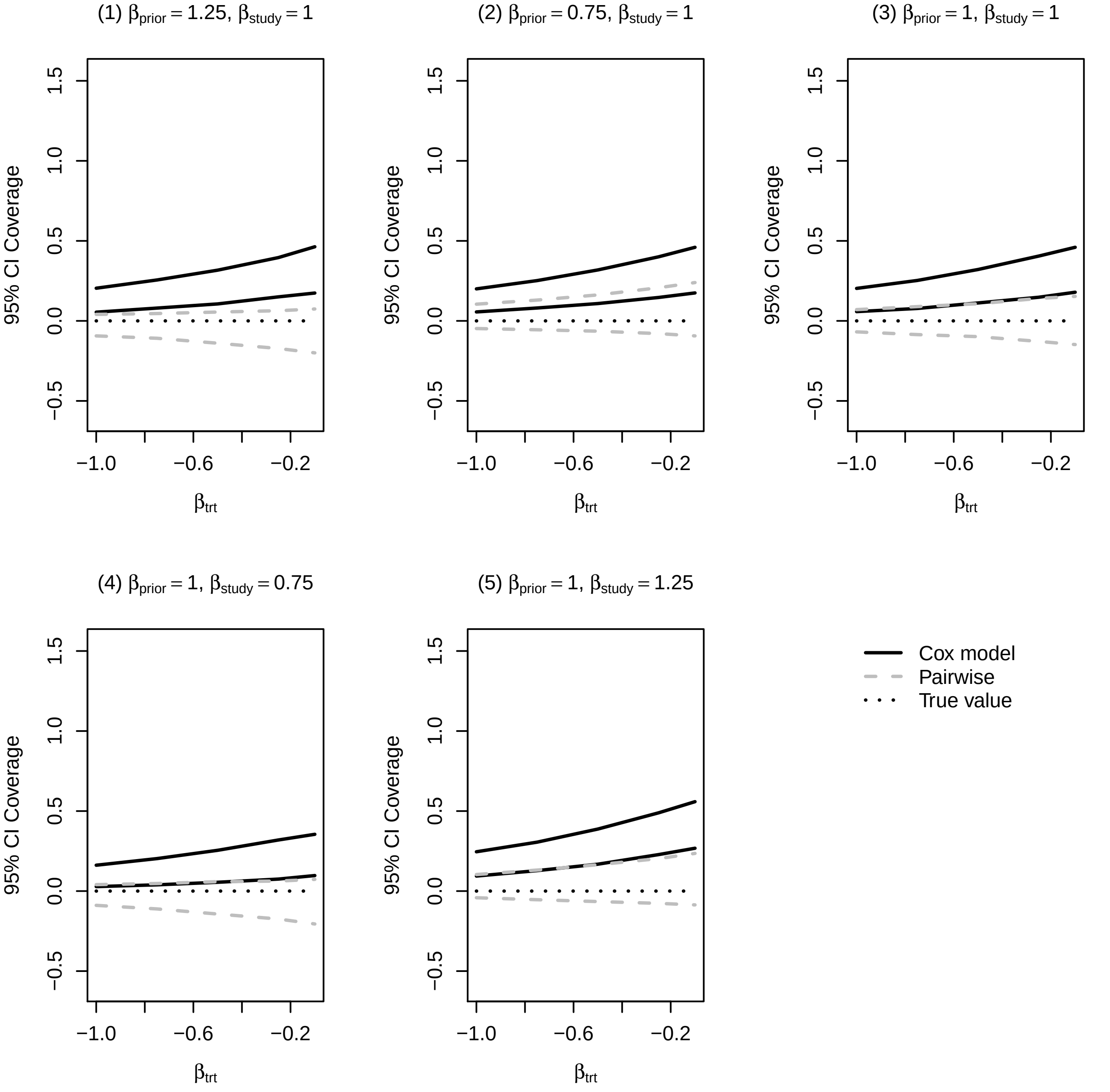

(1) $\beta_{prior} = 1.25$, $\beta_{study} = 1$
(2) $\beta_{prior} = 0.75$, $\beta_{study} = 1$
(3) $\beta_{prior} = 1$, $\beta_{study} = 1$
(4) $\beta_{prior} = 1$, $\beta_{study} = 0.75$
(5) $\beta_{prior} = 1$, $\beta_{study} = 1.25$
95% CI Coverage
β_{trt}
−1.0
−0.6
−0.2
−0.5
0.0
0.5
1.0
1.5
Cox model
Pairwise
True value
