# Supplementary material for: Impact of influenza vaccination on amoxicillin prescriptions in older adults: A retrospective cohort study using primary care data
Source: PLoS One. 2021 Jan 29;16(1):e0246156. doi: 10.1371/journal.pone.0246156 (PMC7846013; doi:10.1371/journal.pone.0246156)
Supplement: S1 Table — (DOCX) [file pone.0246156.s007.docx]

| **Scenario** | **Model assumption** | **Description of scenario** | **Example in the context of our data** | **Parameter values** |
| --- | --- | --- | --- | --- |
| **1** | Treatment and control groups can have different characteristics | Assessing influence of an imbalance in confounder distribution between treatment and control groups | Vaccinated group sicker than control group and additional effects are due to unmeasured confounder | $C_{trt}$ distribution mean:  -0.2, 0.1,0.4, 0.7,1.0 |
| **2** | Confounders must be time-invarying | Change influence of period on unmeasured confounder | Influenza strain in one year has more impact on the frail elderly | $\beta_{prior}$=0.75,1,1.25 ($\beta_{study}$=1)  $\beta_{study}$=0.75,1,1.25 ($\beta_{prior}$=1) |
| **3** | There is no difference in response to treatment within the vaccinated group i.e. no subgroups | A proportion of the vaccinated group do not respond to treatment | Immunosenescence of the elderly, some of the treated group have little to no response to the vaccination | Do not respond to treatment: 0%, 5%, 20%, 35%, 50% |
