# Supplementary material for: Impact of influenza vaccination on amoxicillin prescriptions in older adults: A retrospective cohort study using primary care data
Source: PLoS One. 2021 Jan 29;16(1):e0246156. doi: 10.1371/journal.pone.0246156 (PMC7846013; doi:10.1371/journal.pone.0246156)
Supplement: S2 Table — Figures are relative bias (%) of treatment effect across βtrt from analysis of data simulated from parameters under the three scenarios. (DOCX) [file pone.0246156.s008.docx]

| Scenario | Change | Cox Model  Mean relative bias% (SD) | PERR Pairwise  Mean relative bias% (SD) |
| --- | --- | --- | --- |
| **1** | C_trt_~N(-0.2,0.25^2^) | -25.8(0.2) | 0.3(0.4) |
|  | Ctrt~N(0.1,0.25^2^) | 0.4(0.2) | 0.4(0.2) |
|  | Ctrt~N(0.4,0.25^2^) | 35.4(0.2) | 0.3(0.2) |
|  | Ctrt~N(0.7,0.25^2^) | 82.4(0.4) | 0.2(0.3) |
|  | Ctrt~N(1.0,0.25^2^) | 146.2(0.3) | 0.3(0.1) |
| **2** | β_prior_=1.25, β_study_=1 | 35.2(0.2) | -6.9(0.2) |
|  | β_prior_=0.75, β_study_=1 | 35.1(0.2) | 8(0.2) |
|  | β_prior_=1, β_study_=1 | 35.4(0.3) | 0.5(0.3) |
|  | β_prior_=1, β_study_=0.75 | 25.4(0.3) | -7(0.3) |
|  | β_prior_=1, β_study_=1.25 | 45.9(0.2) | 8.2(0.1) |
| **3** | 0% attenuate | 35.4(0.2) | 0.5(0.2) |
|  | 5% attenuate | 24.4(0.2) | -7.5(0.2) |
|  | 25% attenuate | -1.4(0.4) | -26.5(0.4) |
|  | 35% attenuate | -23.3(0.2) | -42.7(0.1) |
|  | 50% attenuate | -44.9(0.3) | -58.7(0.2) |
