# Supplementary material for: Impact of influenza vaccination on amoxicillin prescriptions in older adults: A retrospective cohort study using primary care data
Source: PLoS One. 2021 Jan 29;16(1):e0246156. doi: 10.1371/journal.pone.0246156 (PMC7846013; doi:10.1371/journal.pone.0246156)
Supplement: S1 File — (DOCX) [file pone.0246156.s009.docx]

**S1 File. Negative Control Outcome.**

N=86637 in unvaccinated group
N=86367 in vaccinated group

N=4042 excluded from analysis because they had had an oedema in the two years prior to 01/09/1999. 1.2% and 1.6% of the unvaccinated group had an oedema in the prior and study periods respectively. 1.7% and 2.3% of the vaccinated group had an oedema in the prior and study periods respectively.

To assess the validity of our estimates using EHR data we analysed a NCO, incidence of oedema (Fig S2). A difference in the risk of oedema was seen in the prior period, HR 1.31 (1.21,1.42); the group who go on to be vaccinated were more likely to experience oedema. This was also seen in the study period, HR 1.37 (1.28,1.47). After adjusting for the prior period with the PERR methods, the artefactual association between oedema and influenza vaccination was eliminated, HR 1.05 (0.95,1.16) and 1.06 (0.95,1.19) for PERR and Pairwise, respectively.
