# Supplementary material for: Impact of influenza vaccination on amoxicillin prescriptions in older adults: A retrospective cohort study using primary care data
Source: PLoS One. 2021 Jan 29;16(1):e0246156. doi: 10.1371/journal.pone.0246156 (PMC7846013; doi:10.1371/journal.pone.0246156)
Supplement: S2 File — (DOCX) [file pone.0246156.s010.docx]

**S2 File. Simulation study.**

**Simulation study design**

The model used for simulation of the prior period was

$${HR}_{Base}exp(\beta_{prior}C_{j})$$

where *C_j_*  represent the unmeasured confounders in group *j*=trt (treatment), ctrl (control). $\beta_{prior}$ is the effect in the prior period of the unmeasured confounders. ${HR}_{Base}$ represents the Weibull distributed baseline hazards. For the study period our models were

${HR}_{Base}exp(\beta_{study}C_{trt}+\beta_{trt}+\beta_{time})$ (1)

${HR}_{Base}exp(\beta_{study}C_{ctrl}+\beta_{time})$ (2)

for the control group (2) and vaccinated group (1). $\beta_{study}$is the effect in the study period of the unmeasured confounders, $\beta_{trt}$ the effect of the vaccination and $\beta_{time}$ a time component.

The baseline hazard distribution was generated to replicate the distribution of the example data and to have similar failure rates. The continuous confounder distributions were $C_{trt}\sim N\left( {0.4,0.25}^{2} \right)$ and $C_{ctrl}\sim N\left( {0.1,0.25}^{2} \right)$ for treatment and control groups respectively. The values for the continuous confounder were based on the range of the propensity scores from our data. The influence of the periods on the unmeasured confounder,$\beta_{prior}$ and$\beta_{study}$, were assumed equal and we added a small time component to the study period, $\beta_{time}=-0.1$. The parameters were chosen to simulate data that approximate that of the CPRD: Treatment effect coefficient, $\beta_{trt}=-0.15$, shape and scale parameter for censored survival times: 10.2, 180, shape and scale parameter for amoxicillin prescription time: 0.95, 3700, shape and scale parameter for death time time: 1.4, 3400. The shape and scale parameters were used to generate the baseline hazards for each patient. Given the parameters $\beta_{prior}$, $\beta_{study},$ $\beta_{trt}$ and $\beta_{time}$ and the confounder distributions $C_{trt}$and $C_{ctrl}$ the survival times were generated for a censored survival time, death time and amoxicillin prescription. The minimum of these times was taken as the outcome for that patient in the prior or study period; patient was censored if the minimum time was the censoring time or death.

A simple power calculation with 5% Type I error, 90% power, equal group allocation and an event probability of 0.05 indicated a minimum sample size of 5200. Simulation sample size was set at 10,000 per group and each parameter set had 1000 repetitions and a fixed random seed.

The relative bias in the treatment effect estimate and confidence interval coverage for the three models using the scenarios and simulation parameters in Table S1 were evaluated.

**Results**

**Scenario 1**

In this set of simulations, we tested the robustness of estimates to differences in the distributions of confounding variables in the treatment and control groups (Table S2, Fig S3). Confounders remain the same in both periods ($\beta_{Prior}= \beta_{Study}=$ 1) but differ between groups, When the treatment and control distributions were the same (Fig S3, panel two) there was no bias and no difference between models, the relative bias was <1% for all models and treatment effects. For a difference in the confounder distributions, the Cox model confidence interval no longer contained the true treatment effect estimate. Relative bias for the Cox model in panel 1 found the treatment effect was underestimated by 25% for all treatment effects and overestimated in panels 3-5 of 35%, 82% and 146%. Relative bias for PERR Pairwise was well controlled and <1% for all cases and treatment effects.

**Scenario 2**

In this scenario, the influence of the period on the confounders was changed (results in Table S2 and Fig S4). The change in the influence of the prior period would have had no effect on the Cox model estimate as it utilises study period data only. When β_study_ reduced the effect of the confounder, the Cox bias decreased (panel 4, relative bias 25%) and when β_study_ increased the influence of the confounder the bias increased (panel 5, relative bias 45%). The imbalance in confounder effect in each period impacted on the PERR Pairwise estimates; the method overestimated the treatment effect when$\beta_{prior}=0.75$ or $\beta_{study}=1.25$ (relative bias 8% overestimate) and underestimated by 7% when $\beta_{prior}=1.25$ or $\beta_{study}=0.75$. The confidence intervals were wider, particularly for small treatment effects, when there was an imbalance between the periods but still contained the true treatment effect.

**Scenario 3**

As the percentage of patients increased who had no treatment effect, PERR Pairwise started to fail, Table S2 and Fig S5. The relative bias for 5%, 20%, 35% and 50% attenuation for PERR Pairwise underestimated the treatment effect by 8%, 27%, 43% and 60%, respectively. The Cox model appeared to be performing better than the PERR methods. However, the bias in the treatment effect estimate seen in the 0% panel is effectively masking the bias induced by the attenuation of the treatment effect.
