# Supplementary material for: Impact of influenza vaccination on amoxicillin prescriptions in older adults: A retrospective cohort study using primary care data
Source: PLoS One. 2021 Jan 29;16(1):e0246156. doi: 10.1371/journal.pone.0246156 (PMC7846013; doi:10.1371/journal.pone.0246156)
Supplement: Attachment — Submitted filename: responsetoreviewers.docx [file pone.0246156.s011.docx]

**Response to editor comments**

*1. Please ensure that your manuscript meets PLOS ONE's style requirements, including those for file naming. The PLOS ONE style templates can be found at*

[*https://journals.plos.org/plosone/s/file?id=wjVg/PLOSOne_formatting_sample_main_body.pdf*](https://journals.plos.org/plosone/s/file?id=wjVg/PLOSOne_formatting_sample_main_body.pdf) *and*

[*https://journals.plos.org/plosone/s/file?id=ba62/PLOSOne_formatting_sample_title_authors_affiliations.pdf*](https://journals.plos.org/plosone/s/file?id=ba62/PLOSOne_formatting_sample_title_authors_affiliations.pdf)

 The paper has been update to conform to PLOS ONE’s requirements.

*2.  Please provide additional details regarding participant consent. In the ethics statement in the Methods and online submission information, please ensure that you have specified (1) whether consent was informed and (2) what type you obtained (for instance, written or verbal, and if verbal, how it was documented and witnessed). If your study included minors, state whether you obtained consent from parents or guardians. If the need for consent was waived by the ethics committee, please include this information.*

*If you are reporting a retrospective study of medical records or archived samples, please ensure that you have discussed whether all data were fully anonymized before you accessed them and/or whether the IRB or ethics committee waived the requirement for informed consent. If patients provided informed written consent to have data from their medical records used in research, please include this information.*

We have added “Patient data received from CPRD is fully anonymised.” to our ethics statement section.

*3. Thank you for stating the following in the Competing Interests section:*

*"I have read the journal's policy and the authors of this manuscript have the following competing interests: WH has a personal long-term shareholding in Glaxo Wellcome, who manufacture an influenza vaccine. WEH has previously received funding from IQVIA."*

*Please confirm that this does not alter your adherence to all PLOS ONE policies on sharing data and materials, by including the following statement: "This does not alter our adherence to  PLOS ONE policies on sharing data and materials.” (as detailed online in our guide for authors* [*http://journals.plos.org/plosone/s/competing-interests*](about:blank)*).  If there are restrictions on sharing of data and/or materials, please state these. Please note that we cannot proceed with consideration of your article until this information has been declared.*

*Please include your updated Competing Interests statement in your cover letter; we will change the online submission form on your behalf.*

*Please know it is PLOS ONE policy for corresponding authors to declare, on behalf of all authors, all potential competing interests for the purposes of transparency. PLOS defines a competing interest as anything that interferes with, or could reasonably be perceived as interfering with, the full and objective presentation, peer review, editorial decision-making, or publication of research or non-research articles submitted to one of the journals. Competing interests can be financial or non-financial, professional, or personal. Competing interests can arise in relationship to an organization or another person. Please follow this link to our website for more details on competing interests:* [*http://journals.plos.org/plosone/s/competing-interests*](about:blank)

 We have updated our competing interests statement:

WH has a personal long-term shareholding in Glaxo Wellcome, who manufacture an influenza vaccine. WEH has previously received funding from IQVIA. This does not alter our adherence to PLOS ONE policies on sharing data and materials. Data are owned by the Clinical Practice Research Datalink (www.cprd.com). Researchers can apply for data access from CPRD and submit a study protocol to the Independent Scientific Advisory Committee for approval.

*4.  We note that you have indicated that data from this study are available upon request. PLOS only allows data to be available upon request if there are legal or ethical restrictions on sharing data publicly. For information on unacceptable data access restrictions, please see* [*http://journals.plos.org/plosone/s/data-availability#loc-unacceptable-data-access-restrictions*](http://journals.plos.org/plosone/s/data-availability#loc-unacceptable-data-access-restrictions)*.*

*In your revised cover letter, please address the following prompts:*

*a) If there are ethical or legal restrictions on sharing a de-identified data set, please explain them in detail (e.g., data contain potentially identifying or sensitive patient information) and who has imposed them (e.g., an ethics committee). Please also provide contact information for a data access committee, ethics committee, or other institutional body to which data requests may be sent.*

*b) If there are no restrictions, please upload the minimal anonymized data set necessary to replicate your study findings as either Supporting Information files or to a stable, public repository and provide us with the relevant URLs, DOIs, or accession numbers. Please see* [*http://www.bmj.com/content/340/bmj.c181.long*](http://www.bmj.com/content/340/bmj.c181.long) *for guidelines on how to de-identify and prepare clinical data for publication. For a list of acceptable repositories, please see* [*http://journals.plos.org/plosone/s/data-availability#loc-recommended-repositories*](http://journals.plos.org/plosone/s/data-availability#loc-recommended-repositories)*.*

*We will update your Data Availability statement on your behalf to reflect the information you provide.*

Thank you. The data are available from CPRD, the authors do not have permission to share the data. We have updated our statement about data sharing to reflect this.

The data used in this study are owned by the Clinical Practice Research Datalink (www.cprd.com). Researchers can apply for data access from CPRD and submit a study protocol to the Independent Scientific Advisory Committee for approval, using the information outlined in the Methods section of the manuscript. The authors had no special access privileges to the data that future researchers would not have.

*5. Your ethics statement should only appear in the Methods section of your manuscript. If your ethics statement is written in any section besides the Methods, please move it to the Methods section and delete it from any other section. Please ensure that your ethics statement is included in your manuscript, as the ethics statement entered into the online submission form will not be published alongside your manuscript.*

The ethics statement has been moved to the end of the Study Cohort section of Materials and Methods

**Response to reviewer comments**

1. *Your outcome of interest (Amoxicillin use) is potentially too vague. Amoxicillin can be used for many different types of bacterial infections. It is not clear why you did not use diagnoses codes for respiratory infections*.

We thank the reviewer for this comment. There is difficulty is selecting an appropriate outcome measure in a study of influenza vaccination using electronic health records; outcomes must be related to the effect of the treatment and well recorded. As noted in the outcomes section of the paper, laboratory confirmed influenza is not well recorded in primary care and all-cause mortality is not considered a useful outcome. Influenza attacks the respiratory system and there is evidence of a reduction in respiratory illness due to influenza vaccination [Gross et al, Nichol et al, Monto et al]. As respiratory illnesses are difficult to diagnose precisely in early stages, patients, particularly the elderly, are often prescribed antibiotics. Antibiotic prescription is well-recorded in routine health records and may serve as a proxy for influenza presenting as a respiratory infection or possible bacterial infection complicating influenza[Kwong et al, Nichol et al, Harper et al]. Our chosen outcome in this study was prescription of amoxicillin, the first choice antibiotic recommended by the National Institute for Health and Clinical Excellence (NICE) for low or moderate severity community acquired pneumonia. Although we could have further qualified our outcome by only selecting amoxicillin prescriptions where there was an associated clinical code for respiratory illness, we chose to use a more sensitive definition in this exploratory study to maximise available sample size. Our choice of within-patient design (based on the PERR method) should ensure that we remove the effect of any differences in levels of amoxicillin prescription for non-respiratory infections between vaccinated and unvaccinated groups. Text has been added to the Material and Methods Outcomes section to clarify our choice of outcome measure. Relevant amoxicillin codes were identified by a pharmacist. We acknowledge the limitations of our study in paragraph 3 of Strengths and Limitations section of the Discussion and agree further work should investigate this outcome measure further.

Gross PA, Hermogenes AW, Sacks HS, Lau J, Levandowski RA. The efficacy of influenza vaccine in elderly persons. A meta-analysis and review of the literature. Ann Intern Med 1995;123:518–27
Nichol KL, Treanor JJ. Vaccines for seasonal and pandemic influenza. J Infect Dis 2006;194 Suppl:S111–8. doi:10.1086/507544.
Monto AS, Hornbuckle K, Ohmit SE. Influenza vaccine effectiveness among elderly nursing home residents: a cohort study. Am J Epidemiol 2001;154:155–60.
Kwong JC, Maaten S, Upshur RE, Patrick DM, Marra F. The effect of universal influenza immunization on antibiotic prescriptions: an ecological study. Clin Infect Dis 2009;49:750–6. doi:10.1086/605087
Harper SA, Fukuda K, Uyeki TM, Cox NJ, Bridges CB. Prevention and control of influenza. Recommendations of the Advisory Committee on Immunization Practices (ACIP). MMWR Recomm Rep 2005;54:1–40.

2. *Would also have used time-varying cox regression to assess vaccination status. You potentially have introduced immortal time into the vaccinated cohort.*

Although immortal time bias could be a potential problem with the PERR methodology as, by design, patients must be alive at the start of the study period, this issue has been addressed by previous authors including Tannen et al (2009) in response to a comment on their original paper. As with their original study design, our patients in both exposure groups had a predefined time interval to meet entry criteria for the study (no vaccination for two years prior to the study period). There was a large pool of unvaccinated patients (n=171274) who were matched to vaccinated patients based on the characteristics in Table 1. Their start date for the study period was effectively random as it was allocated by their matched vaccinated counterparts. Immortal time bias is introduced when the period of “immortality” before exposed patients receive treatment is either misclassified with regards to treatment status or excluded from the analysis. Neither of these issues apply here and Tannen et al point out that immortal time bias should not be an issue due to the careful choice of design. Furthermore, we have previously conducted a simulation study that showed the Pairwise PERR method is unbiased in the presence of differential case fatality because of the within-patient nature of the comparisons (Lin and Henley, 2016). We also note that the PERR framework set out by Lin and Henley (2016) allows for flexible modelling including use of multiple time-varying covariates and effects (Section 7.1). The version of the Pairwise PERR model that we use in this study does indeed allow for the time-varying effect of vaccination status.

Tannen Richard L, Weiner Mark G, Xie Dawei. Use of primary care electronic medical record database in drug efficacy research on cardiovascular outcomes: comparison of database and randomised controlled trial findings BMJ 2009; 338 :b81

Lin NX, Henley WE. Prior event rate ratio adjustment for hidden confounding in observational studies of treatment effectiveness: a pairwise Cox likelihood approach. Stat Med. 2016 Dec 10;35(28):5149–69.

3. *Would be nice to see the covariates you matched on. Did you control for senility?*

Subjects were matched on the covariates in Table 1. This has been clarified in Material and Methods Study Cohort section. We have matched on dementia to control for senility (Table 1).

4. *You mention health seeking behaviour. Can you think if any proxies that you could use to account for health seeking behaviour?*

We have tried to capture health seeking behaviour through including the number of GP visits over the 12 months of the prior period as a covariate. We have used the number of consultations within one year as a snap shot of health seeking behaviour and assumed this to be representative during our study. However, we acknowledge that a change in consultations over this time period may be indicative of changing health. A limitation of current PERR methodology is the assumption that any unmeasured confounders are time invariant. We have added to the discussion this limitation (paragraph two of strengths and limitations) and highlighted the need for further work.
